# Return to work a bumpy road: a qualitative study on experiences of work ability and work situation in individuals with chronic whiplash-associated disorders

**DOI:** 10.1186/s12889-021-10821-w

**Published:** 2021-04-23

**Authors:** A. Peolsson, A. Hermansen, G. Peterson, E. Nilsing Strid

**Affiliations:** 1 Department of Health, Medicine and Caring Sciences, Unit of Physiotherapy, Linköping University, Linköping, Sweden; 2 Centre for Clinical Research Sörmland, Uppsala University, Uppsala, Sweden; 3 University Health Care Research Center, Faculty of Medicine and Health, Örebro University, Örebro, Sweden

**Keywords:** Whiplash injuries, Neck pain, Occupational health, Return to work, Qualitative research

## Abstract

**Background:**

Work resumption is a big challenge in the rehabilitation process for individuals with whiplash-associated disorders (WAD). To better meet the needs of individuals with WAD in their return to work process, more knowledge on their experiences and perspectives is needed. The aim of this study was to explore the experiences of work ability and the work situation of individuals who participated in a neck-specific exercise programme for chronic WAD.

**Methods:**

This qualitative study has an exploratory and descriptive design based on data collected through open-ended interviews with 17 individuals with chronic WAD. Data were analysed inductively using conventional content analysis.

**Results:**

Analysis of the data yielded the following five categories related to the participants’ narratives on their experiences of work ability and their work situation: *Return to work – a process of setbacks and bureaucracy*; *The need to be understood by health care professionals, and to receive a treatment plan*; *Individual resources are important for work ability*; *The consequences of reduced work ability*; and *Working conditions are important for work ability*.

**Conclusion:**

Individuals with chronic WAD often struggle to return to work. Emotional and practical support from stakeholders is imperative and needs to be strengthened. Participating in a neck-specific exercise programme, including being acknowledged and receiving information about WAD, could positively affect the work ability of WAD sufferers. This study has provided management strategies to improve the ability to work for individuals with chronic WAD, and highlights the need to incorporate a healthy and sustainable return to work in the rehabilitation of individuals with WAD, thereby making their return to work a success.

**Supplementary Information:**

The online version contains supplementary material available at 10.1186/s12889-021-10821-w.

## Background

Individuals with chronic whiplash-associated disorders (WAD) experience a variety of symptoms, including pain and disability, leading to financial consequences for themselves, their employer, and society [1–3]. These individuals return to work more slowly, and their return to work rate is lower, compared with individuals sick-listed with other musculoskeletal disorders [3, 4]. Approximately 50% of those with WAD who return to work experience ongoing pain and disability [5], which may affect their work ability [6]. Poor work ability is related to the personal factors higher age, neck pain, multiple pain locations, cognitive dysfunction, low health-related quality of life, and pessimistic illness perceptions [7–10] as well as work-related stress and work dissatisfaction [7]. Circularly associations have been found between pain relief, functional improvement, and improvement in work capacity (measured as working hours/week) [11]; however, there are few studies evaluating the effect of rehabilitation on work ability for individuals with chronic WAD, and thus far the effect is inconclusive [12–14]. Adams et al. reported only marginal improvement in work ability despite rehabilitation [12]. Work ability was, however, improved after a multi-professional rehabilitation programme with cognitive behavioural therapy [13] and after neck-specific exercises with and without a behavioural approach [14].

Previous qualitative studies have explored the experience of living with WAD [15–18], including challenges in returning to work [18–20]. Work resumption was identified as the biggest challenge in the rehabilitation process [19] and in one study, participants expressed sadness over being unable to work [20]. There are however no previous studies focusing on work ability and work situation from the perspective of individuals with chronic WAD. Since the effect of rehabilitation on work ability is inconclusive [12–14], more knowledge of the individuals’ experiences is needed. This knowledge could be used to better meet the needs of individuals with WAD in their return to work process. The aim of this study was to explore the experiences of work ability and the work situation of individuals who participated in a neck-specific exercise programme for chronic WAD. In this study, we use the definition of work ability as the balance between an individual’s resources and work demands [21].

## Methods and materials

### Design

This qualitative study has an exploratory and descriptive design based on data collected through open-ended interviews [22]. Data were analysed inductively using conventional content analysis according to Hsieh & Shannon [23].

### Setting and participants

Participants were recruited from an ongoing randomized controlled trial (RCT) [24] evaluating two different ways of distributing neck-specific exercises to individuals with chronic WAD Grade II and III [25] in primary health care in Sweden. Both groups performed neck-specific exercises for 12 weeks: Arm A received internet-based support exercises in combination with four visits to the physiotherapist and arm B received exercises at a physiotherapy clinic 2 times/week. Those who had completed at least half of the training sessions and the 1-year follow-up were eligible to participate in the present study. A purposeful sampling strategy was used to achieve a heterogeneous sample of participants from both RCT arms based on age, gender and geographical area, in order to obtain the richest possible data [22]. Out of 72 eligible individuals, 44 were purposefully approached, ten at a time according to the purposeful sampling strategy, and received brief written information about the study and an interest request through a text message by one of the researchers. Twenty-one responded and received extended oral and written information as well as a consent form. Interviews were continuously held with those who returned informed consent until informational power [26] was deemed to have been achieved. In total, 17 individuals were included, comprising 13 women and four men. The participants were between the ages of 25 and 61 years and came from eight different geographical areas. Details of the participants are provided in Table 1. At the time of the interviews, the majority of the participants (16 out of 17) had returned to ordinary or modified work full or part-time. Since the time of the car accident, a few had been requested to re-train for a new job, had changed jobs by themselves or had been entitled to benefits from the Social Insurance Agency (SIA). One person was waiting for work capacity evaluation. In Sweden, decisions on entitlement to sickness benefits is made by the SIA primarily based on information provided by physicians in a sickness certificate.

**Table 1:** Characteristics of the participants in the time of the interview

Age, mean (range) years^a^	47 (24–60)
Female gender, n (%)ª	13 (75%)
Duration post whiplash injury in months, mean (range)^a^	28.5 (8–54)
Present neck pain intensity, VAS 0–100 mm, mean (range)	29.9 (0–73)
Neck-specific disability, NDI % score 0–100, mean (range)	34 (4–56)
Number of participants working^a^, n (%)	16 (94%)
White collar workers^a^, n (%)	12 (75%)
Current work ability, single item WAI 0–10, mean (range)	6.6 (1–10)
RCT group A, n (%)ª	65%

Range = minimum to maximum; NDI, Neck Disability Index, % score 0 (no disability) to 100 (complete disability); VAS, Visual analogue scale, score 0 - (no pain) to 100 (worst possible pain); WAI, work ability index and the single item current work ability compared with the lifetime best, score 0 (completely unable to work) to 10 (work ability at its best); RCT group A refers to the allocation in the randomized controlled trial, receiving internet-based exercise support and few physiotherapy visits

^a^Baseline data, otherwise 1 year after the end of the rehabilitation program

### Data collection

A semi-structured interview guide with open-ended questions was developed by the authors concerning the participants’ experiences and perceptions of their work ability and work situation, from the accident until the time of the interview, and anything they had experienced as facilitating or hindering their ability to work. To deepen the discussion, probing questions related to the participants’ narratives were posed, such as “Could you elaborate that?” The interview guide was pilot-tested in one interview with a patient with WAD but not participating in the RCT. The interview was not analysed, but only minor revisions were made. An English version of the interview guide is provided as supplementary information (Additional file 1). As the participants were spread over a large geographical area, the interviews were conducted by phone. The interviews were conducted in October–December 2019 by one of the authors (E.N.S.), who was not involved in the design of the ongoing RCT. The interviews were digitally recorded. Each interview lasted between 45 and 60 min. The interviewees were pseudonymised and each assigned a number. A professional transcriber transcribed the interviews verbatim. The transcripts were not returned to participants for comments.

### Data analysis

Data were analysed using qualitative conventional content analysis [23]. The analysis was data-driven [23] and based on the participants’ unique experiences. Two of the authors (E.N.S. and A.P.) were responsible for the analysis and held continuous discussions throughout the analytical steps. The interview text was thoroughly read twice to gain an understanding of the whole [23]. Initial impressions were written down, and then discussions were held and comparisons made of the understanding of the text [23]. The transcribed interview text was imported into NVivo 12 (QSR International, Melbourne, Australia), which was used to manage and code data. Meaning units related to the aim of the study were identified and coded for their content by A.P. The two main authors (E.N.S and A.P) discussed the codes and worked together to sort related codes into meaningful clusters forming the basis for developing subcategories, which were finally grouped into a smaller number of categories [23]. The subcategories and categories were compared for differences and similarities, striving to be internally homogeneous and externally heterogeneous, which means that no data should fall between two groups nor fit into more than one group [22]. The process was iterative, going back and forth between the main text and the codes. The consistency and adequacy of the subcategories and categories was checked by a third author (A.H.) and finally discussed among all four authors. Quotations capturing the essence of what had been said were selected to illustrate the different subcategories. The selected quotations were translated into English by a professional language translator and then retranslated into Swedish, to ensure that the meaning was retained. Finally, all authors discussed the categorization and the selected quotations and reached consensus. The coding tree is described in Table 2.

**Table 2:** Coding tree. Examples of meaning units, codes, subcategories and categories from content analysis of interviews with individuals with whiplash-associated disorders

Meaning unit	Code	Subcategory	Category
Then I could recover over the weekend so I could cope with work again on Monday. But it was a really hard time. (Participant 5)	Recover over the weekend to cope with work, hard times.	1.1 The bumpy road to return to work	1. Return to work – a process of setbacks and bureaucracy
One tried to work as hard as usual, 100%, as I did the whole time. But that did really not work. (Participant 7)	Working as usual did not work		
This job was good for me when I started it (…) but then this happened and I have to re-think (…) there must be a change because one is not getting any younger either. If I would continue doing this (job), the risk of more symptoms would be pretty high. I really think that. (Participant 8)	To continue with the job increases the risk for symptom aggravation	1.2 Work motivation and confidence in future work ability	
I do believe that I can continue work like this until I retire. I will have problems, pain that is, but it doesn’t matter what kind of job I have, because I think I’ll have to live with this pain, simple as that. But I got used to the idea and it works fine. And with these work tasks, absolutely, it works. (Participant 6)	Pain will persist but I can live with it and be able to work until retirement		

### Ethical considerations

Participation in the current interview study was voluntary. Participants were interviewed after giving oral and written informed consent. All data were decoded and handled with confidentiality as they contained information about the participants’ health. Only the research team had access to the interview files, transcripts and consent forms. Participants were assured that no individual would be identifiable from the quotations or results. In order to maintain confidentiality, no information on age, gender or other participant characteristics were ascribed to the quotations. The interviewer had had no previous contact with the participants. The study followed the ethical principles of the Helsinki Declaration (World Medical Association 2019) and was approved by the Regional Ethics Review Board in Linköping, Sweden (Peolsson, Dnr 2016/135–31 and 2018/462–32).

## Results

Analysis of the data from the 17 interviews yielded five categories related to the participants’ narratives on work ability and the work situation before and after participating in a neck-specific exercise programme for chronic WAD: *Return to work – a process of setbacks and bureaucracy*; *The need to be understood by health care professionals, and to receive a treatment plan*; *Individual resources are important for work ability*; *The consequences of reduced work ability*; and *Working conditions are important for work ability*. Each category is supported by two to three subcategories (Table 3).

**Table 3:** Overview of categories and subcategories describing the participants’ experiences of their work ability and work situation

Category	Subcategory
*1. Return to work – a process of setbacks and bureaucracy*	1.1 The bumpy road to return to work
	1.2 Work motivation and confidence in future work ability
	1.3 Conforming to social insurance regulations
2. *The need to be understood by health care professional, and to receive a treatment plan*	2.1 The neck-specific exercise programme – a tool for better work ability
	2.2. Being on sick leave – not a stand-alone treatment
3. *Individual resources are important for work ability*	3.1 Individual strategies to handle work demands
	3.2 Planning, prioritizing and recovering in spare time – putting all energy into work
	3.3 Emotional and practical support from relatives
4. *The consequences of reduced work ability*	4.1 Changed self-image and work role
	4.2 Poor work ability affects the workplace and the company, and participants’ financial situation
5. *Working conditions are important for work ability*	5.1 Manager support and workplace adjustments
	5.2 Emotional and practical support from colleagues

### Return to work – a process of setbacks and bureaucracy

The category *Return to work – a process of setbacks and bureaucracy* encompasses the process the participants underwent from the accident to the interview 1 year after participating in the neck-specific exercise programme, including setbacks such as being on-and-off work. During this process, participants had varying degrees of self-esteem and confidence in their future work ability and at the same time had to face challenges in dealing with the bureaucracy and learning about sickness insurance regulations.

#### The bumpy road to return to work

All participants expressed a willingness to return to work quickly and not to be on sick leave. The return to work was a bumpy process which included setbacks and going on- and-off work because of variations in symptoms, and which took enormous amounts of energy. Some participants had returned to ordinary work tasks immediately after the accident or after a short period of sick leave; others gradually returned to work after a longer sick leave period; but the return to work was uniformly described as a difficult struggle.

*... it’s getting better and better. I can’t say it’s actually good now, but it’s much better than it was at the beginning, and getting to this point has been a real struggle, that’s for sure ... (Participant 6)*

#### Work motivation and confidence in future work ability

The participants described being strongly motivated to return to working life. Work was part of their identity and they valued it highly. It was important to return to work as soon as possible after the accident and to stay at work, even when symptoms impacted on their work ability – as there were no other options. The thought of being on sick leave was frightening because the participants were afraid of both symptom aggravation and negative mood due to being at home and off work. They strived to lead an ordinary life where work was central. Work could trigger positive emotions such as pride and happiness, give meaningfulness and energy, and distract from pain and worry.

*It’s incredibly important to me to have a job - that much I’ve realized. I don’t know if I could stand having to go on long-term sick leave. So somehow I have to have a job to make my life seem worthwhile. (Participant 7)*

The participants described a variety of expectations regarding their future work ability, from having confidence in their ability to sustain or increase their work hours despite symptoms and being optimistic about future work ability, to feelings of hopelessness and worries about aggravated symptoms due to work demands or ageing affecting their future work ability and participation in working life. To regain a sense of hope, thoughts of changing job were described; however, this was perceived as impossible by some because they felt they lacked the education or were too old for the labour market.

*The only thing I could do is to actually change (jobs). Or I could work part-time with this and then have another at like 25% so I could have another job to go to. But, it’s not easy to change (jobs) when you’re starting to … I’m 56, goodness. There are 40-year olds that can’t get a job (laugh). No, but I’m actually thinking of doing something else. (Participant 8)*

#### Conforming to social insurance regulations

The participants described their encounters with the SIA in largely negative terms. It was difficult to navigate the insurance system, and participants described that they were being pressured by the SIA to return to work too fast. This generated feelings of distrust and a need for the participants to defend themselves, which could be a struggle. It was also stressful to wait for the SIA’s decisions on entitlement to sickness benefits. The participants hesitated to apply for sickness benefits based on these experiences, which may imply that they conformed to the regulations in the Swedish insurance system but with varying degrees of acceptance. Instead of being on sick leave, some participants chose to work part-time with subsequently reduced income.

*... I’ve reduced my hours at my own request. I haven’t had the energy to deal with this and keep on fighting, and stuff ... (Participant 8)*

Others had to continue working, and felt distrusted by the SIA:

*… when I had this last whiplash injury, the SIA didn’t seem to understand that I had become worse, so I didn’t receive any benefits for that last injury. It feels like they think you’re just making it up, and that’s also the reason why one keep struggling, so you can get by and have a bearable life, financially so to speak (Participant 7)*

#### The need to be understood by health care professionals, and to receive a treatment plan

The importance of being understood by health care professionals with knowledge about their disorder and of receiving a treatment plan was highlighted in the interviews. After participating in a neck-specific exercise programme, the participants described having a tool for better work ability: they had gained increased knowledge about WAD, had a stronger neck and fewer symptoms, and could manage work tasks more efficiently than before.

#### The neck-specific exercise programme – a tool for better work ability

After participating in the study with neck-specific exercise programme, the participants described reduced WAD-related symptoms, which contributed to being able to perform work tasks more efficiently and to work more hours than before. Examples of increased work ability were being able to participate in meetings without supporting the head with their hands or leaning against a wall, being able to drive a car, and staying focused at their computer. Especially the exercises were highlighted as important for work ability:

*It is the exercises. I really ‘ve to keep doing my exercises for my neck and back. I just cannot give it up, because it affects both my work and private life. (Participant 6)*

The increased knowledge, gained from the programme and the physiotherapists involved, was used as a tool at work to reduce, handle or counteract WAD-related symptoms so they would not interfere with the work. The physiotherapist had also confirmed their symptoms and diagnosis, which, together with the knowledge and exercises, contributed to the participants feeling empowered. To participate in the study with neck-specific exercises was described as a turning point:

*So then I participated in this study, and then after, once I’d done that, it was as if I somehow got my life back. It was completely different. I could suddenly do my job - I could perform - without losing my concentration, and so on. And also, I didn’t feel the pain so much. (Participant 9)*

#### Being on sick leave – not a stand-alone treatment

Previous contacts with health care had mostly resulted in short-term sick leave, pain medications and physiotherapy treatments that were perceived as not very effective, such as acupuncture, manual therapy or different exercise treatments. The participants felt that there was insufficient knowledge about WAD among health care professionals: sometimes the participants felt distrusted, and most of all they lacked care continuity and a treatment plan. As described by the participant in the quotation below, receiving a sickness certificate is not sufficient.

*… it would have been much easier if you’ve had someone who understood, or had the knowledge of me and my situation. But when you see different doctors every time, then it’s hard (…) if you’ve seen ten doctors, then you’ve ten different assessments, and I’ve never tried to get a sick note, because I’ve just one goal and that is to work and feel as well as possible. So, I’ve seen how some doctors – they think that I should go on sick leave for several weeks or months and that’s the end of it. But that won’t help me get any better. (Participant 12)*

### Individual resources are important for work ability

The participants described different strategies to handle work demands, ranging from strategies at work to planning their leisure time, and the emotional, practical or financial support they received from their family. They prioritized work and adjusted and planned the rest of life in order to manage work demands.

#### Individual strategies to handle work demands

To handle work demands, the participants used a variety of strategies they either developed themselves or had learned from the neck-specific programme. These helped them prevent or manage symptoms so they would not interfere with the ability to work. The strategies included standing at the computer or desk, taking short breaks, and keeping an upright posture with a long neck, which enabled them to sit for a longer time, even when driving a car. Flexibility in work time, working from home and digital participation in meetings were other strategies they used. The participants emphasized the importance of being able to set reasonable work conditions for themselves by setting priorities, avoiding stress and working at their own pace. Other strategies employed were to use a scarf around the neck, take pain medication, withdraw from social activities at work, or just to bite the bullet and carry on regardless. Short-term or part-time sick leave, or working part-time without financial support was the last resort when the pain interfered too much with the ability to work. However, it was not easy to choose between these strategies and to know what may the best strategy in the long end:

*And then, I guess, one has to try at work that – will it get better if I sit down, will it get better if I stand? Would it be better if I go home for the day, or can I handle this, and is there another way to solve this? One has to try a little. But it’s not easy – it really isn’t – I wouldn’t say. (Participant 10)*

#### Planning, prioritizing and recovering in spare time – putting all energy into work

In order to manage work demands, the participants continuously had to plan and adapt their leisure time because of their symptoms. They prioritized rest and recovery, which often meant avoiding or reducing social activities with family and friends. Although they acknowledged that leisure time and physical activity were important and contributed to their ability to work, they said that the activities had to be planned and balanced. Feeling trapped by their symptoms made it difficult to plan activities or act spontaneously. Altogether, most energy was put into work:

*I have to schedule time for recovery and even beforehand - whenever I go and teach, for example, I have to schedule a quieter day the day before. So I still have to schedule things according to what I manage. (Participant 1)*

#### Emotional and practical support from relatives

The participants described how their relatives provided emotional support by showing understanding, and being attentive and caring. Relatives further provided encouragement and set boundaries regarding the participants’ activities. Participants who were single parents described the challenges of managing work demands and having full responsibility for the household and family, which often impacted on their ability to be physically active and perform exercises. Practical support, in the form of housework, from family members was a prerequisite for managing work demands:

*I have to restrict my daily life all the time to manage work. I cannot do any housework; I don’t do anything at home just to manage go to work. I do no housework or anything – my son has been doing the cooking and cleaning for 5½ years now, so that I can go to work. (Participant 14)*

### The consequences of reduced work ability

The experience of reduced work ability affected the participants in terms of altered self-image and work role, and the participants were also concerned about the impact of their work disability on both their workplace and the company, and their own financial situation.

#### Changed self-image and work role

The participants expressed sadness over changed self-image and work role when they were not able to perform work tasks as they had previously done, which they sometimes perceived as a defeat. They told of how it was difficult to handle feelings of not recognizing oneself and being dispensable at work, for example when one’s work tasks were being performed by colleagues. Struggling with a lowered self-image was most prominent during the first period after the accident, but even later, the participants continued to struggle to re-orient themselves and cope with the new situation. Highlighting the value of work, one participant said:

*So I had a well-paid, enjoyable, stimulating job that actually largely made me who I was – it developed my personality, gave me an enormous amount of experience and opportunities to meet fantastic people. And as if by magic, there I was, suddenly stuck at home on long-term sick leave. It’s the kind of sudden reversal of fortune that could make anyone very depressed. What’s more, everything is now so financially precarious – everything’s uncertain. So apart of constant pain and fatigue, the truth is you also lose many things you value that existed for you before. (Participant 16)*

#### Poor work ability affects the workplace and the company, and participants’ financial situation

The participants described being on long-term sick leave or working part-time as a catastrophe, both from a mental and from a financial perspective. It could result in becoming financially dependent on one’s parents or partner. They recognized that their work disability affected not only themselves, but also their workplace, in terms of reduced productivity because they no longer had the same working capacity and had to slow down their working pace. Colleagues’ work could also be hindered and the general workflow slowed when participants were unable to perform their work tasks in time. When the participants had a managerial position and did not have the capacity to run a company, this had a distressing impact on the financial situation of the company, as well as on the employees. Informants also expressed that their colleagues had to take on more responsibility for work tasks to, and fill in for them.

*it is stressful for me when someone else must do my work tasks. It’s not like nobody needs to do the tasks, instead it is someone else who has to do them. And then, something else will be affected. (…) and I know that she (my colleague) will do her work tasks in addition to my tasks (Participant 14)*

Participants described pain and insufficient sleep, which led to subsequent concentration problems, tiredness and bad temper, affecting colleagues and others, such as patients or students:

*What happened just before I went on sick leave is that I was sitting and looking at the student who was sitting across from me and telling me about his problems, and I was thinking, “I wonder if he’ll notice if I nod off for a minute now?” - Because I was so tired. (Participant 4)*

### Working conditions are important for work ability

Having work with workplace flexibility and physical and psychosocial demands the participants deemed as acceptable for themselves, facilitated their ability to work. Also, feeling understood and acknowledged, and receiving practical support, such as workplace adjustments, from colleagues and the manager was imperative.

#### Manager support and workplace adjustments

Work ability was facilitated by flexibility at work based on the participants’ specific needs. The participants appreciated when they were given a variety of work tasks with acceptable physical and psychosocial demands, as well as independence at work. They described, however, a range of experience of support from their managers. Having an open communication with one’s manager, who shows understanding and empathy, and feeling needed at the workplace even when symptoms affect one’s work ability, was described as emotionally supportive. Decisions on workplace adjustments, such as ergonomic, adjustable furniture or devices; a change of work tasks or working hours; and changes to the participants’ work position were made on the initiative of the manager or in dialogue with the participants, and sometimes with support from occupational health services or Human Resource Management and Development (HRMD) departments.

*I said I couldn’t keep on working in this department; I had to, I started to look for other jobs [...] Then my boss thought that was no good – I couldn’t be applying for other jobs because I couldn’t work. So they created a new position for me. [...] So I am in more of an instructor role now [...]. It’s thanks to my boss that they wanted to keep me on. (Participant 6)*

All of this practical and emotional support was perceived as helpful and improved the participants’ ability to work. Lack of support, engagement and understanding from managers, by contrast, was described as disappointing.

*During that first year after this injury had occurred, it would have been good for me if perhaps my manager had asked some more. One spends a lot of time at work, that’s how it is, so these contacts one has at work there are very important. (Participant 13)*

#### Emotional and practical support from colleagues

The participants described the importance of trust and support from colleagues. However, in order to receive support, they had to be open themselves about their impairments and difficulties in performing their work tasks. Colleagues do care and provide both emotional and practical support by taking responsibility for heavy work tasks, rotating work tasks and taking a co-worker’s work disability into account. The participants said that it was important to have somebody at work to chat with, and to share one’s negative thoughts and emotions such as worry.

*That the entire workforce [...] and my boss, you know, that they really asked how I’m doing, and how my neck is now. [...] They can keep on asking like this now, I mean, about like: “How’s it going? I’m wondering about you now with your neck.” So it’s extremely ... I have an extremely good workplace, I must say. People care about each other and that has meant so much, obviously. It makes you want to go to work. (Participant 9)*

On the other hand, participants mentioned that they sometimes felt questioned, and then they withdrew from others. They worried about whether or not their colleagues might feel unfairly treated when their (the participant’s) adjusted work tasks meant more work for them (the colleagues). The support from colleagues in some cases decreased over time, as the work disability continued.

*So, I’ve simply been given the easier work tasks. I definitely did. And that’s not really popular among (co-workers), others must think it’s unfair of course, I get that. (Participant 14)*

## Discussion

This study highlights how individuals with chronic WAD struggle to return to work, and reflects some of the sadness the participants expressed over a changed self-image and work role. The participants were motivated to work and had developed a range of strategies to handle work demands. They related that having emotional and practical support from managers, co-workers, family, health care professionals and others was important; however, their experiences of support varied. Participating in a neck-specific exercise programme, and thus being acknowledged and receiving information about WAD, positively impacted their work ability. These findings will be further discussed in relation to the developmental and dynamic process of return to work [27] and the holistic model of work ability [21].

The focus of this study was on work ability and the work situation from the perspective of individuals with chronic WAD. As suggested in the developmental process of return to work, workers pass through a series of phases when returning to work, including the experience of injury or chronic illness, being off work, and re-entering work, as well as maintenance of work ability and advancement at work [27]. The participants in this study described a bumpy and uphill road to return to work, which included setbacks and going on-and-off work. This supports the developmental and dynamic process of return to work described by Young and colleagues, [27] that workers move non-linearly between the mentioned phases. The findings underscore that individuals with chronic WAD need to feel understood and acknowledged by their managers, colleagues, family, health care providers and other stakeholders during this process. According to previous studies, this bumpy road may be explained by different strategies to cope with fluctuations in symptoms, but also by poor self-efficacy [28], a mismatch between stakeholder expectations [29, 30] and/or insufficient support from health care [17, 19, 31], the SIA [17, 19] or employer [32, 33]. Our findings show how the support for return to work for individuals with chronic WAD can be strengthened.

As anticipated from the holistic work ability model [21], a balance between the human resources (the individual’s health and functional capacities, competence, values, attitudes, and motivation) and work (demands and content of work, work environment, community, and management) is crucial. A poor balance between human resources and work decreases the person’s work ability. In addition, the balance is also affected by the environment outside of work, such as family and the close community [21]. The narratives from the participants in this study support that health and functional capacity are an important dimension affecting (though not altogether determining) work ability. Participating in a neck-specific exercise programme could mean receiving a tool for managing symptoms at work, being able to perform work tasks more efficiently and working more hours.

These results are in line with previous findings from quantitative data on symptom reduction [34] and improved work ability [14] after neck-specific exercises. In contrast to previous experiences with health care providers, the participants in our study also described feeling understood and acknowledged by the physiotherapists involved in the study. This meant that their injury was “legitimized”; they received information and a treatment plan; and the exercises reduced their symptoms. This type of acknowledgement may be related to the term “validation”. As suggested by Linton [35], validation in pain communication functions to soothe negative affect by acknowledging the patient’s experience and thereby increasing disclosure to promote problem solving and shared decision making. The importance of being understood by health care professionals [15], and of receiving information and support to understand and cope with one’s situation, has previously been highlighted in qualitative studies in individuals with WAD [13, 16, 17, 19]. Feeling believed and getting validation of the whiplash injury is considered a necessary step in the recovery process [17], with implications for return to work as suggested by the findings in this study. According to a previous meta-analysis, better management strategies to support return to work and daily life in general are needed for individuals with WAD [18].

Human resources as described previously [21, 36] consist also of a person’s inner values and attitudes as well as factors that motivate them in their working life. A wish to participate in working life has previously been described by individuals with WAD [19, 31]. In this study, the participants all expressed a strong motivation to work. Work was an important part of their identity and they put a lot of effort into maintaining their working life. They described balancing between different strategies to handle work demands using active and passive coping strategies to handle their symptoms. This pattern is in accordance with other studies on patients with WAD [31], emphasizing the need to unravel self-efficacy beliefs, emotions, coping strategies, as well as expectations to better support individuals with WAD in the process of returning to work [18]. The aforementioned holistic model of work ability suggests that, within the dimensions of work, management and leadership have the strongest effect on work ability. Managers are suggested to play a key role in influencing the balance between the resources of the individual and work by organizing the work according to the requirements and capabilities of the individual [36]. Sustainable return to work after musculoskeletal disorders and common mental disorders is influenced by an interplay of multiple factors, among which the most consistent evidence was found for support from leaders and co-workers [33]. The importance of emotional and practical support, in terms of timely work adjustments and ergonomics, from the manager was highly emphasized in the interviews as contributing to participants’ ability to work, as was also the importance of being part of a working environment with occupational health services, a good psychosocial climate, and co-worker support. However, not everyone was given this support. These findings highlight the importance of strengthening a supportive communication between the manager, co-workers and the individual during the return to work process.

Our findings suggest that, with better health care support, including validation, information, and an individually tailored, neck-specific exercise programme and treatment plan, individuals with WAD can be empowered and their return to work can be less difficult. This means, to be listen to and believed by the stakeholders involved, and to receive evidence-based rehabilitation. The rehabilitation of individuals with WAD could be improved by implementation of the neck-specific exercise programme in primary health care, education of physiotherapists, physicians and other health care professionals along with information about WAD to media and various stakeholders such as head of health care departments, patients, SIA, workplaces and HRMD departments. Setting return to work as a priority in the rehabilitation of individuals with WAD is strongly recommended in the literature [37]. As acknowledged in the model of work ability [21], in previous research [10, 37, 38] as well as in this study, symptom reduction is important but it is not a prerequisite for work ability and successful work integration. Rather, work ability is affected by the balance between the individual’s resources and work as well as the environment outside of work. This study has provided management strategies to support the ability to work for individuals with chronic WAD, and suggestions on how to incorporate a healthy and sustainable return to work in the rehabilitation of individuals with WAD, thereby helping to make the process smoother.

## Methodological considerations

This study has some strengths and limitations, which need to be considered when interpreting the results. Credibility was demonstrated by the fact that the research team had good scientific knowledge and clinical experience of treating patients with WAD, and of sick leave and return to work, as well as previous experience in conducting qualitative studies. The interview guide was pilot-tested, which further strengthens credibility. The sample size was guided by information power [26]. A recruitment of 15–20 participants was planned, and during the research process a sample of 17 participants were deemed to be sufficiently large and varied to elucidate the aim of the study and contribute to new knowledge. We included more women than men, which is consistent with the gender distribution in chronic WAD [2]. In contrast to the 1:1 ratio in the RCT, slightly more participants from treatment arm A were included. Considering other background data such as age, gender and duration since whiplash injury the current cohort was deemed to be representative to the RCT (unpublished data). The interviews were held over the phone, allowing recruitment of participants from different geographical areas in Sweden. Telephone interviews may allow participants to feel relaxed and speak freely, but to not see and respond to the informants’ facial expressions could hamper the data quality by for an example less in-depth interviews. The data were systematically analysed using conventional content analysis and keeping the analysis close to the text [23]. To strengthen confirmability, the entire research team held consensus discussions throughout the analysis process, and the findings, including supporting quotations, were approved by the research team. No member check was however performed. The researchers are all physiotherapists and all are women. We have been aware of our perspectives during the data collection and analysis, and have strived to correctly convey the participants’ perspectives in the results. The checklist for reporting of qualitative studies [39] was used to improve transferability. The findings from this study may serve as a starting point for further research on strategies to support the return to work process among individuals with WAD from the perspective of the individual, workplace and health care provider.

## Conclusion

Individuals with chronic WAD often struggle to return to work. Emotional and practical support from stakeholders is imperative to the success of return to work and needs to be strengthened. Participation in a neck-specific exercise programme, including acknowledgement and information about WAD, could positively affect the work ability of WAD sufferers. This study has suggested management strategies to support the ability to work for individuals with chronic WAD and highlights the need to incorporate a healthy and sustainable return to work in the rehabilitation of individuals with WAD, thereby making the return to work a success.

## Supplementary Information

**Additional file 1.**

## Data Availability

The data generated and analysed during the current study are governed by the legal provisions of Linköping University, Sweden. The datasets generated and analysed during the current study are not publicly available owing to the sensitive and personal nature of the data, according to Swedish Data Protection Law, but will be available from the corresponding author on reasonable request.

## Abbreviations

HRMD: Human Resource Management and Development department
NDI: Neck disability index
RCT: Randomized controlled trial
SIA: Social Insurance Agency
VAS: Visual analogue scale
WAI: Work ability index
WAD: Whiplash-associated disorders
