# Supplementary material for: Return to work a bumpy road: a qualitative study on experiences of work ability and work situation in individuals with chronic whiplash-associated disorders
Source: BMC Public Health. 2021 Apr 23;21:785. doi: 10.1186/s12889-021-10821-w (PMC8067290; doi:10.1186/s12889-021-10821-w)
Supplement: Supplementary file 1 — Additional file 1. [file 12889_2021_10821_MOESM1_ESM.docx]

**Interview guide**

How would you describe your experience with working since the car accident?

How would you describe your ability to work in relationship to your whiplash symptoms?

How do you manage symptoms related to your whiplash injury if they flare up at work?

Can you think of any specific factors at your place of work that improves, or enables, your ability to work?

Can you think of any specific factors at your place of work that decreases, or hinder, your ability to work?

Are there any outside factors that affect your ability to work?
